# Low-temperature extracts of Purple blossoms of basil (*Ocimum basilicum L.*) intervened mitochondrial translocation contributes prompted apoptosis in human breast cancer cells

**DOI:** 10.1186/s40659-020-00324-0

**Published:** 2021-01-06

**Authors:** Mariam Abdulaziz Alkhateeb, Wedad Refaiea Al-Otaibi, Qwait AlGabbani, Amena Ali Alsakran, Alaa Ahmed Alnafjan, Amani Mohammed Alotaibi, Wedad Saeed Al-Qahtani

**Affiliations:** 1 Department of Biology, College of Science, Princess Nourah Bint Abdulrahman University, Riyadh, Saudi Arabia; 2 Department of Biology, College of Sciences and Humanities, Prince Sattam Bin Abdulaziz University, Al-Kharj, Saudi Arabia; 3 King Saud Medical City, Riyadh, Saudi Arabia; 4 Department of Forensic Sciences, College of Criminal Justice, Naif Arab University for Security Sciences, P.O. Box 6830, Riyadh, 11452 Saudi Arabia

**Keywords:** Basil (*ocimum basilicum l.*), Purple blossoms, Aqueous extract at low temperature (0 °C/6 h), Mitochondrial fission, Apoptosis, Human breast cancer cells (MCF7), Anticancer

## Abstract

**Background:**

The preventive and therapeutic medical utilization of this plant is an age-long practice across the globe. This study aimed to validate the impact of dark purple blossoms of basil (*Ocimum basilicum L.*) aqueous extract at low temperature (0 °C) mediated mitochondrial fission contributed to induced apoptosis in human breast cancer cells.

**Methods:**

Fresh blossoms were extracted at low temperature (0 °C) using a watery solvent. Human MCF7 breast cancer cells were then treated with 3 separate fluctuated concentrations of 0, 50, 150 and 250 µg/mL for 24 and 48 h.

**Results:**

The outcomes demonstrated the presence of anthocyanins, anthraquinones, tannins, reducing sugars, glycosides, proteins, amino acids, flavonoids and volatile oils and nonappearance of Terpinoids and alkaloids. Contrastingly, frail presence of steroids in basil blossoms aqueous concentrate was noted. In addition, the results from a phytochemical subjective examination of basil (*Ocimum basilicum L.*) blossoms aqueous extract demonstrated that most of the credited natural impacts containing more remarkable contents of antioxidants and anticancer compounds in basil blossoms aqueous extract. Moreover, the restraint of glucose take-up was alleviated mediated by a dose-dependent manner in MCF7 cells with basil (*Ocimum basilicum L.*) blossoms aqueous extract inducted for 24 h, resulting in mitochondrial fission.

**Conclusion:**

This is the first study that shows the impact of the aqueous extract of basil (*Ocimum basilicum L.*) blossoms was extracted at low temperature (0℃/6 h) underlined high amounts of flavonoids and phenolic compounds bearing more anticancer and antioxidant activities compared to another aqueous extract (using boiled water solvent) and alcoholic extracts.

## Background

*Ocimum basilicum L.* (Basil) (Lamiaceae family)-a famous culinary herb, develops in numerous regions globally [1]. Basil is a society restorative plant, acknowledged authoritatively in various nations [2, 10]. Today, it is assessed that 80% of the total populace depends on plant arrangements as medication to meet their wellbeing needs [3, 29]. Basil (*Ocimum basilicum L.*) is a plant that has a place with the family Labiatae and has demonstrated its capability to help deflect a few ailments in different nations. Numerous examinations have built up that basil leave activities have intense cancer prevention agents, curbs aging, is an anticancer, antiviral, and has antimicrobial properties [4–10]

Nevertheless, there is no report accessible for the bioactive parts of basil (*Ocimum basilicum L.*) blossoms aqueous extract at low temperature (0 °C) acquired from species developed in Abha, Saudi Arabia just as their pharmacological activities on the fission of the mitochondria contribute prompted apoptosis in human breast malignant growth cells. Hence, the present investigation was aimed at explaining the impact of the chemical composition of basil (*Ocimum basilicum L.*) blossoms aqueous extract in Abha, Saudi Arabia.

## Materials and methods

### Preparation of aqueous suspension and plant extracts

**Fig. 1:**
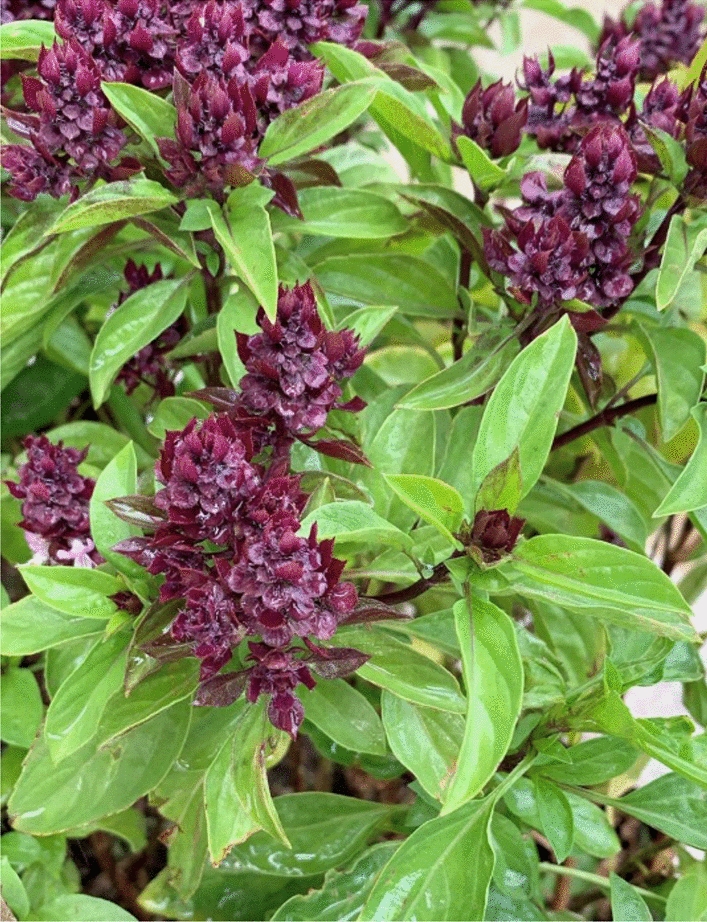
Dark purple basil (*Ocimum basilicum L.*) blossoms were utilized in the current study, Abha, Saudi Arabia

Dark purple basil (*Ocimum basilicum L.*) blossoms were gathered from the southern area of Saudi Arabia, Abha, (Fig. 1) and (Table 1). Fresh blossoms were cleaned under running water and squashed then blended in deionized water (1:20 w/v), followed extraction at a low temperature (0 ℃/6 h). Rough concentrate was centrifuged at 3000 rpm for 15 min. The aqueous plant extract was lyophilized utilizing vacuum freeze-dryer (Model FDF 0350, Korea). The gooey powders acquired were then reconstituted in the correct volume to get a stock arrangement, of a centralization of 50 mg/mL. The stock arrangement was safely stored at 4 °C until utilized. The yield amount for the concentrate was discovered 12.8% per 100 g of the blossoms.

**Table 1:** Description of plant under study

Parameter	Basil
Botanical name	*Ocimum basilicum L.*
Family	Lamiaceae
English name	Basil, Saint-Joseph's-wort
Vernacular name (in Arabian region, KSA)	Rehan
Part used	Blossoms
Checked names on Plant list http://www.theplantlist.org;	http://www.theplantlist.org/tpl1.1/search?q= Ocimum basilicum L.

### Phytochemical qualitative analysis

The subjective phytochemical investigation was done for the test of saponins (Foam test), reducing sugars, tannins test, steroids, glycosides, volatile oils alkaloids, flavonoids, proteins as well as amino acids and terpenoids (Salkowki's Test) as per [11, 12].

### Assessment of anthocyanins and anthraquinones

*Ocimum basilicum L.* blossoms aqueous extract was screened for carried anthocyanins and anthraquinones according to standard methods reported by (Abduljalil's Test) as per [13].

### Total phenolic content determination

The summative phenolic substance of *Ocimum basilicum L.* blossoms aqueous extract was dictated by Folin-Ciocalteu spectrophotometric technique (Hamad's Test) as per [14].

### HPLC conditions for polyphenolic compounds quantification

Measurement of phenolic compounds of *Ocimum basilicum L.* blossoms aqueous extract was resolved using High-Performance Liquid Chromatography (HPLC) as indicated by (Zordoky's Test) as per [15]. Ten phenolic gauges of phenolic mixes were utilized: gallic corrosive, caffeic corrosive, coumaric, syringic corrosive, vanillin, cinnamic corrosive and pyrogallol, catechin, quercetin and rutin. Agilent 1260 interminability HPLC arrangement (Agilent, USA), outfitted with quaternary siphon, a Zorbax Eclipse in addition to C18 segment 100 mm × 4.6 mm i.d., (Agilent innovations, USA) worked at 25 °C, was utilized for phenolic compound examination. The infused volume was 20μ: VWD finder set at 284 nm. The division is accomplished utilizing a ternary straight elution slope with (A) HPLC grade 0.2% H3PO4 (v/v), (B) methanol and (c) acetonitrile. The infusion volume for Ocimum basilicum L. blossoms aqueous extracts was 1 g/10 mL. All principles were broken up in ethanol and infused with the accompanying concentrations; gallic = 12 μg/mL, caffeic corrosive = 12 μg/mL, coumaric corrosive = 8 μg/mL; syringic corrosive = 8 μg/mL, vanillin = 8 μg/mL, cinnamic corrosive = 4 μg/mL, pyrogallol = 65 μg/mL, catechin = 40 μg/mL, quercetin = 32 μg/mL and rutin = 40 μg/mL. Mixes were recognized by contrasting their maintenance times and UV–Vis spectra with those of the norms, while their concentrations were determined relying upon the region under the pinnacle of models.

### Cell culture conditions

Human MCF7 breast cancer cell line was bought from the American Type Culture Collection (ATCC, Manassas, VA, USA) and kept in DMEM/Ham's F-12 (1:1 v/v) medium enhanced with 100 mL/L FBS, 1.5 g/L sodium bicarbonate, 400 µg/mL hydrocortisone, 10 mL/L penicillin and streptomycin (zero.1 mg/mL).

### Cell treatment

From the humidified hatchery, cells seeding was additionally done at 1 × 10^6^ cells/well or 1 × 10^5^ cells/well in 96 well tissue culture plates separately. An aqueous extract from (*Ocimum basilicum L.*) blossoms was put on to a culture media and the cells were then treated with 3 separate fluctuated concentrations of 0, 50, 150 and 250 µg/mL for 24 and 48 h.

### Direct counting method

Once the treatment steps were accomplished, cells were mixed with 20 μL of phosphate-buffered saline (PBS) for each well per 96 wells in the plate. The cultured cells were trypsinized and then have used a trypsinized drop of cells from every well of the 96 wells plate into a haemocytometer using a pipette to measure the cell viability. Living cells were looked round and transparent, and the dead cells were dense and shrunk. Percentage of viability of the cells was obtained using the formula below:

$$(\% )\;{\text{Cell}}\;{\text{viability}} = \frac{{{\text{Absorbance}}\;{\text{of}}\;({\text{treated}}\;{\text{and/or}}\;{\text{exposed}})\;{\text{viable}}\;{\text{cells}}}}{{{\text{Absorbance}}\;{\text{of}}\;({\text{untreated}}\;{\text{and/or}}\;{\text{unexposed}})\;{\text{viable}}\;{\text{cells}}}}.$$

### Measurement of glucose uptake

2-[N-(7-nitrobenz-2-oxa-1,3-diazol-4-yl)amino]-2-deoxy-D-glucose (2-NBDG) was used to treat MCF7 cells for 30 min, after aqueous extract of (*Ocimum basilicum L*.) blossoms-treatment for 24 h. MCF7 breast cancer cells glucose uptake was assayed using flow cytometry (Becton–Dickinson, San Jose, CA).

### Determination of (ROS)-Reactive Oxygen Species Production

Subcellular ROS was examined fluorometrically by estimating the of a non-fluorescent test 2,7-dichlorofluorescein diacetate (DCF-DA) oxidation to a fluorescent metabolite dichlorofluorescein (DCF) via mitochondrial ROS just as depicted beforehand with slight adjustments [30]. Gathered cells were suspended in 500 mL of PBS and mixed in with 10 mM (last centralization) of dichloro-dihydro-fluorescein diacetate (DCFH-DA) for 20 min at 37 °C. The cells suspension were deposited at 1200 rpm for 5 min. Therefore, the cells were washed thrice with 500 mL of Phosphate-Buffered Saline (PBS)/ pellet to evacuate excess DCFH-DA. The ROS level was tested by flow cytometry (Becton–Dickinson, San Jose, CA).

### RNA extraction and cDNA synthesis

All out RNA was separated utilizing Invitrogen-TRIzol reagent as indicated by the maker's guidelines and evaluated by estimating the absorbency at 260 nm. The quality of RNA was controlled by estimating 260/280 proportions. From that point, the synthesizing of the cDNA-strand was produced utilizing the High-Amplitude cDNA turn around interpretation pack (Applied Biosystems) as indicated by the maker's directions [16].

### Measurement of mRNA expressions by real-time polymerase chain reactions (RT-PCR)

The primers were utilized in the present examination (Table 2) were bought from (Invitrogen, USA). Measure controls were consolidated in separated wells but onto a similar plate, to be more specific. All the samples and controls were run in triplicates on an ABI 7500 Fast Real-time PCR. The quantitative RT-PCR data was breaking down by a near edge (Ct) strategy, and the overlap acceptances of treated examples were contrasted and the untreated examples. Relative quality expression (i.e., ΔΔCT) strategy as earlier outlined was used to analyse the data on the RT-PCR [17]. *β-actin* was utilized as an interior reference gene to standardize the declaration of the selected genes.

**Table 2:** Primers sequences utilized for real-time PCR reactions

Gene	Reverse primer	Forward primer
CASPASE-3	CCTCACGGCCTGGGATTT	GAGTGCTCGCAGCTCATACCT
p53	GGGAGAGGAGCTGGTGTTG	GCCCCCAGGGAGCACTA
BCl2	GCCGGTTCAGGTACTCAGTCA	CATGTGTGTGGAGAGCGTCAA
DR4	GTGCTGTCCCATGGAGGTA	AGTACATCTAGGTGCGTTCCTG
HO-1	TGTTGCGCTCAATCTCCTCCT	ATGGCCTCCCTGTACCACATC
NQO1	CGTTTCTTCCATCCTTCCAGG	CGCAGACCTTGTGATATTCCAG
β-actin	GGCATAGAGGTCTTTACGGATGTC	TATTGGCAACGAGCGGTTCC

### Determination of Caspase-3 activity

Activities of Caspase-3 were calorimetrically estimated via the CaspACE testing framework acquired from Biovision (Mountain View, California, USA) in respect to the producer's guidelines. Succinctly, the MCF7 cells overlaid to 12-well cell cultured plate were treated for 24 h with numerous concentrations of basil blossoms extract. Subsequently, the MCF7 cells were afterwards gathered via trypsinization and the cell pellets were suspended in cold cell lysis cushions and were ice-treated for 1\6 h. The acquired supernatant through centrifugal force for 1\6 h at 10,000×*g* at 4 °C was then placed in a Fresh cylinder and later stored at − 20 °C. For purposes of caspase-3 action estimating, approximately 30 μg protein matter was cultured under 200 μM compound clear-cut, acetyl-Asp-Glu-Val-Asp *p*-nitroanilide, colorimetric caspase-3 substrate I (Ac-DEVD-pNA) at a temperature of 37 °C for 120 min. Caspase-3 actions were evaluated via estimation of absorbance at 405 nm with a reader plate (Bio-Tek Instruments, Winooski, VT).

### Mitochondrial fission

Cells were exposed for 30 min using 250 nM of MitoTracker Deep-Red FM (Invitrogen) in a free culture. After two time-washing with PBC, nuclei were recolored by Hochest 33342 for 10 min. A microscope (Zeiss LSM700 confocal) was used to view the mitochondrial morphology.

### Statistical analysis

Analytical examinations were performed by use of SigmaStat programming adaptation 3.5 (Systat Software, San Jose, CA, USA). Quantitative outcomes were presented as mean standard deviations. Esteems of p being lower than 0.05 were deemed statistically imperative.

## Results

### Phytochemical qualitative analysis

Table 3 showed the phytochemical subjective investigation of basil (*Ocimum basilicum L.*) blossoms aqueous extract. The outcomes demonstrated the presence of anthocyanins, anthraquinones, tannin, reducing sugars, glycosides, amino acids, flavonoids and volatile oils and nonappearance of Terpinoids and alkaloids. Contrastingly, frail nearness of steroids in basil blossoms aqueous extract was noted.

**Table 3:** Phytochemical screening of basil (*Ocimum basilicum L.*) blossoms aqueous extract profile

Phytochemical	Extract content
Anthocyanins	+
Anthraquinones	+
Reducing sugars	+
Tannins	+
Glycosides	+
Alkaloids	−
Flavonoids	+
Volatile oils	+
Amino acids/proteins	+
Terpinoids	−
Saponins	+
Steroids	+

Trace content presence (+), Trace content absence (−)

### Phenolic and flavonoids profile

Absolute flavonoids and phenolic content of basil (*Ocimum basilicum L.*) blossoms aqueous extract are delineated in (Table 4). Absolute phenolic content (TPC) and complete flavonoid content (TFC) of basil scored (186.31 and 34.25 mg/g) individually.

**Table 4:** Total flavonoids and phenolic contents in basil (*Ocimum basilicum L*.) blossoms watery extract profile

Test	Concentration (mg/g)
Total phenolic content (TPC)	186.31 ± 2.41
Total flavonoids content (TFC)	34.25 ± 1.87

### HPLC phenolic compounds quantification

Table 5 stood for phenolic compounds examination of basil (*Ocimum basilicum L.*) blossoms aqueous extract using HPLC. Among nine tried norms quercetin was > vanillin > cinnamic > coumarin corrosive which demonstrated the most noteworthy concentrations (2.529, 4.062, 2.322 and 2.456 μg/mL respectively), while gallic and syringic acids recorded less scores (0.32 and 1.41 μg/mL respectively), rutin and caffeic acids recorded trace concentrations (0.0015 and 0.015 μg/mL respectively), as catechin was not recognized.

**Table 5:** Phenolic compounds investigation of basil (*Ocimum basilicum L.*) blossoms aqueous extract by means of HPLC examination

Phenolic compound	Concentration (μg/mL)
Gallic acid	0.3242 ± 0.021
Catechin	ND
Syringic acid	1.417 ± 0.24
Caffeic acid	0.0158 ± 0.058
Rutin	0.00156 ± 0.0017
Coumarin	2.456 ± 0.018
Vanillin	4.0624 ± 0.013
Quercetin	2.529 ± 0.26
Cinnamic acid	2.3221 ± 0.026

*ND* not detected

### Effect of (*Ocimum basilicum L.*) blossoms aqueous extract on MCF7 Cell proliferation

**Fig. 2:**
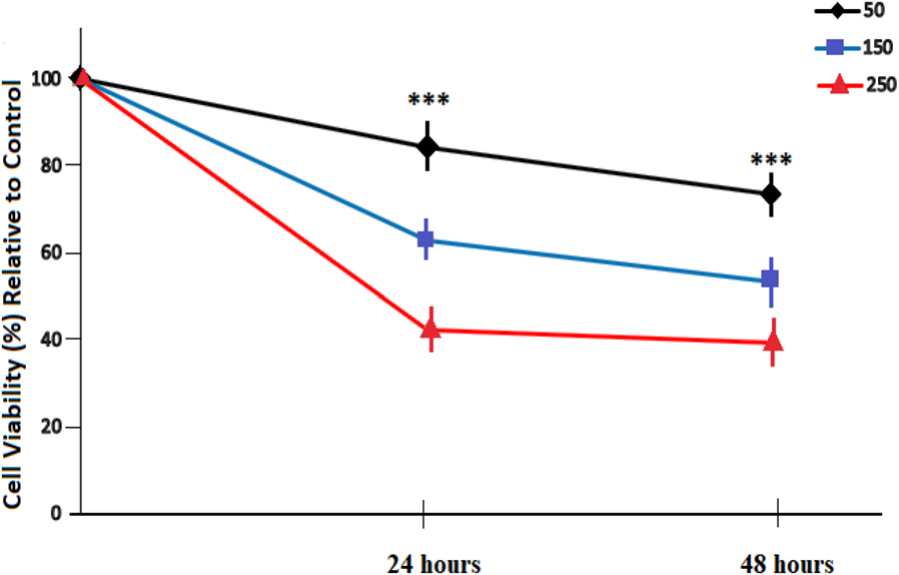
Effect of basil (*Ocimum basilicum L.*) blossoms aqueous extract on MCF7 cells viability and growth. Cells were put in incubation with several basil (*Ocimum basilicum L*.) blossoms concentrations for 24 and 48 h, after which cell growth was ascertained utilizing direct counting tests. Values are put as percentages of the control (mean ± SEM, n = 5) ***P < 0.001, **P < 0.01, *P < 0.05 in comparison to the control (0 µg/mL)

To determine the ability of basil (*Ocimum basilicum L.*) blossoms aqueous extract to inhibit growth and proliferation of cancer cells, MCF7 cells were treated with steadily increasing concentrations of basil (*Ocimum basilicum L.*) blossoms aqueous extract (0, 50, 150, and 250 µg/mL) for 24 and 48 h, after which cell reasonability and expansion were determined using Direct counting strategy. Figure 2 exhibits that endurance of MCF7 cells were altogether diminished after incubation with basil (*Ocimum basilicum L.*) blossoms separate in a focus subordinate way when contrasted with untreated MCF7 cells (Fig. 2), proposing that basil (*Ocimum basilicum L.*) blossoms aqueous extract is tumor cell selective. The determined IC_50_ for basil (*Ocimum basilicum L.*) blossoms watery extract is around 250 µg/mL.

### Inhibition of glucose uptake and mitochondrial activity

**Fig. 3:**
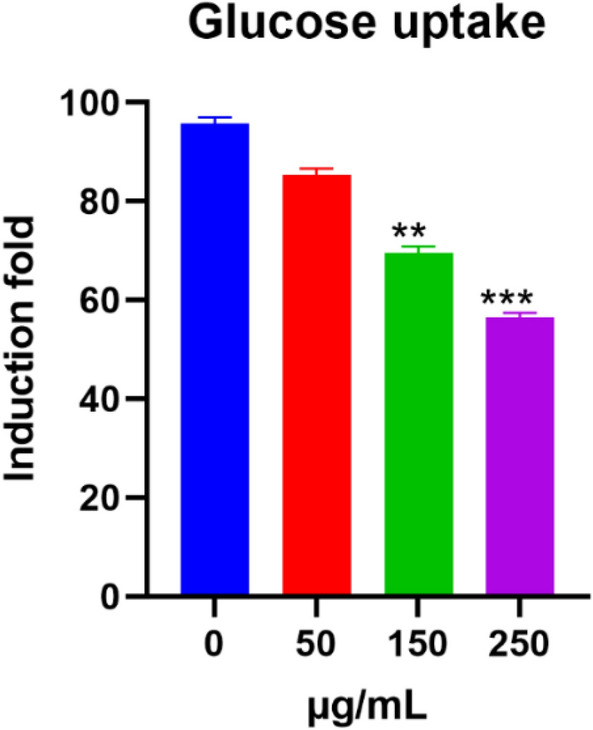
Restraint of glucose take-up in MCF7 cells that were treated by basil (*Ocimum basilicum L.*) blossoms aqueous extract inducted for 24 h. The glucose take-up was measured by flow cytometry. Data were recorded as mean ± SEM, (n = 5) ***P < 0.001, **P < 0.01, *P < 0.05 contrasted with control (0 µg/mL)

**Fig. 4:**
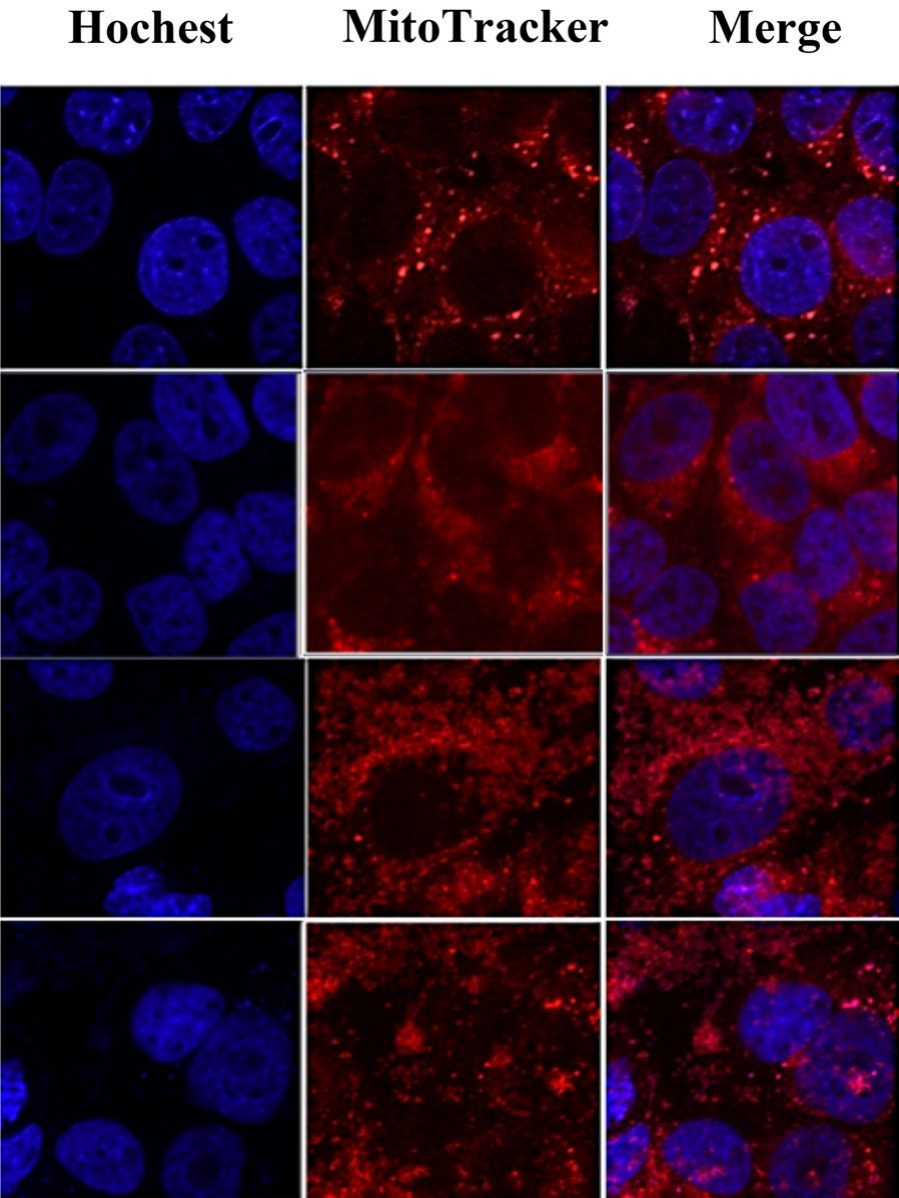
The MCF7 cells showing pro-apoptotic, abnormal shape of nuclei (Hochest 33342-blue stain) and mitochondrial fusion (MitoTracker-Deep Red stain) in all treated cells by basil (*Ocimum basilicum L.*) blossoms aqueous extract (50, 150, and 250 µg/mL), for second, third and fourth raw respectively. While the first raw represent normal nuclei shape with mitochondrial morphology of MCF7 cells (0 µg/mL). The photographs were seen under microscope (confocal)

During glucose metabolism, ATP production and cell proliferation, both are significant in cell growth. Nevertheless, if glucose absorption was inhibited there is a subsequent of cell growth suppression. We discovered that the uptake of glucose (2-NBDG) uptake was affected by basil blossoms aqueous extract. The glucose take-up restraint was eased by a dose-subordinate way in MCF7 cells with basil blossoms aqueous extract treatment for 24 as shown in (Fig. 3). The mitochondrial morphology was changed by basil blossoms aqueous extract treatment, mitochondrial splitting was seen in basil blossoms aqueous extract treated groups for 24 h (Fig. 4).

### Effect of (*Ocimum basilicum L.*) blossoms aqueous extract on the expression of oxidative stress genes and ROS production in MCF7 cells

**Fig. 5:**
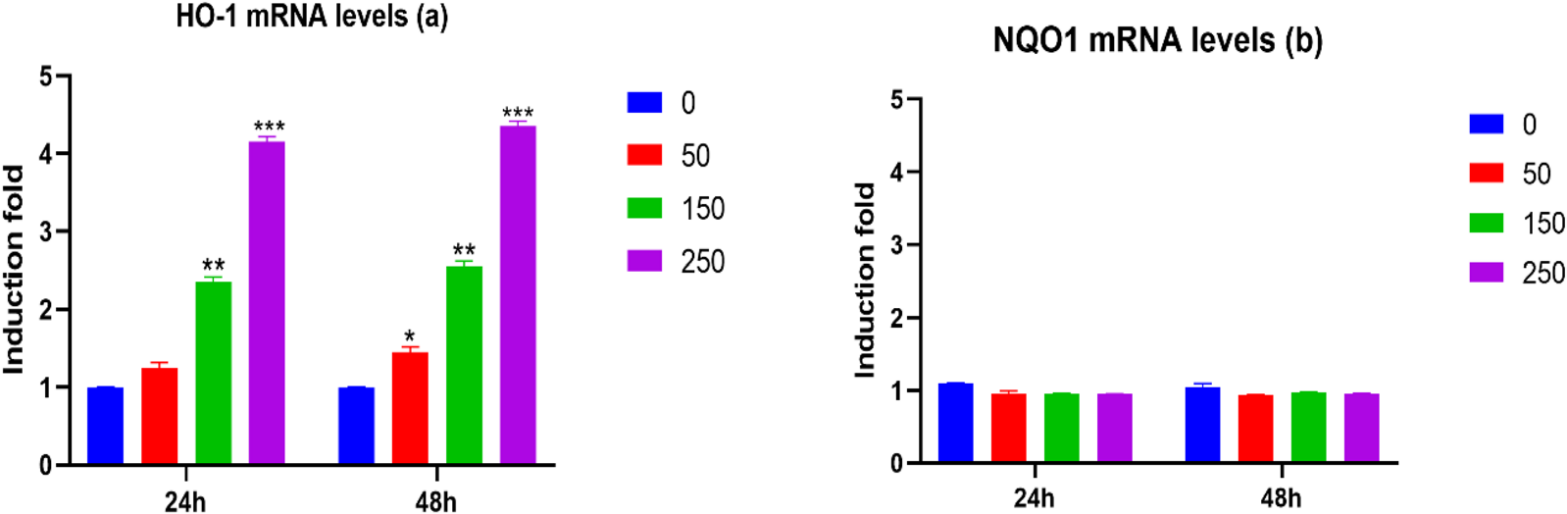
Effect of basil (*Ocimum basilicum L.*) blossoms aqueous extract on oxidative pressure markers *HO-1* (**a**) and *NQO1* (**b**) mRNA levels in MCF7 cells treated for both 24 and 48 h with different concentrations of basil (*Ocimum basilicum L.*) blossoms aqueous extract (0, 50, 150, and 250 µg/mL). From there on, the mRNA levels of *HO-1* and *NQO1* were measured utilizing RT-PCR and standardized to *β-actin* housekeeping gene. Data were recorded as means ± SEM (n = 5) of three free investigations. ***P < 0.001, **P < 0.01, *P < 0.05 contrasted and untreated cells

**Fig. 6:**
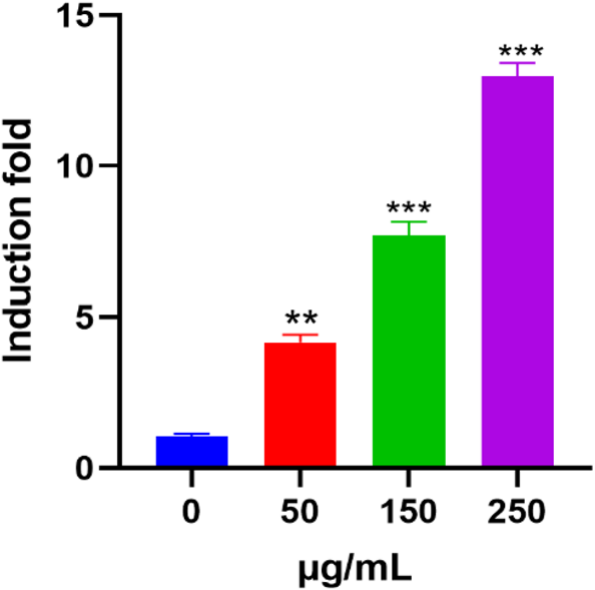
ROS creation in MCF7 cells was treated for 24 h with different groupings of basil (*Ocimum basilicum L.*) blossoms aqueous extract (0, 50, 150, and 250 µg/mL). From that point, cells were treated with c (10 μM) for one h. DCF arrangement was estimated fluorometrically utilizing excitation/outflow frequencies of 484/535 nm. Values were introduced as means ± SEM, (n = 10). ***P < 0.001, **P < 0.01, *P < 0.05 contrasted with control (0 µg/mL)

To examine if basil (*Ocimum basilicum L.*) blossoms aqueous extract interceded oxidative stress, we determined the capacity of (*Ocimum basilicum L.*) blossoms aqueous extract to balance the declaration of oxidative pressure genes in human breast cancer cells, MCF7 cells. Consequently, MCF7 cells were treated with same convergence of (*Ocimum basilicum L.*) blossoms aqueous extract for 24 and 48 h, from that point ROS creation and *NQO1* and *HO1* mRNA levels were estimated by DCF and RT-PCR measure, respectively. Our outcomes demonstrated that (*Ocimum basilicum L.*) blossoms aqueous extract (Fig. 5), fundamentally initiated *HO-1* mRNA levels in MCF7 cells in a fixation subordinate way (Fig. 5a) nonetheless; actuated *HO-1* mRNA levels just at the most noteworthy concentrations tried (150 and 250 µg/mL) for 24 h and (50, 150 and 250 µg/mL) for 48 h. Interestingly, (*Ocimum basilicum L.*) blossoms aqueous extract did not adjust the statement of *NQO1* mRNA levels (Fig. 5b), while it essentially expanded the ROS creation at all concentrations tried in a fixation subordinate way (Fig. 6) with a most extreme acceptance of 5, 8 and 12-overlays accomplished by 50, 150 and 250 µg/mL of (*Ocimum basilicum L.*) blossoms aqueous extract, respectively.

### Effect of (*Ocimum basilicum L.*) blossoms aqueous extract on the mRNA expression level of apoptotic genes in MCF7 Cells

**Fig. 7:**
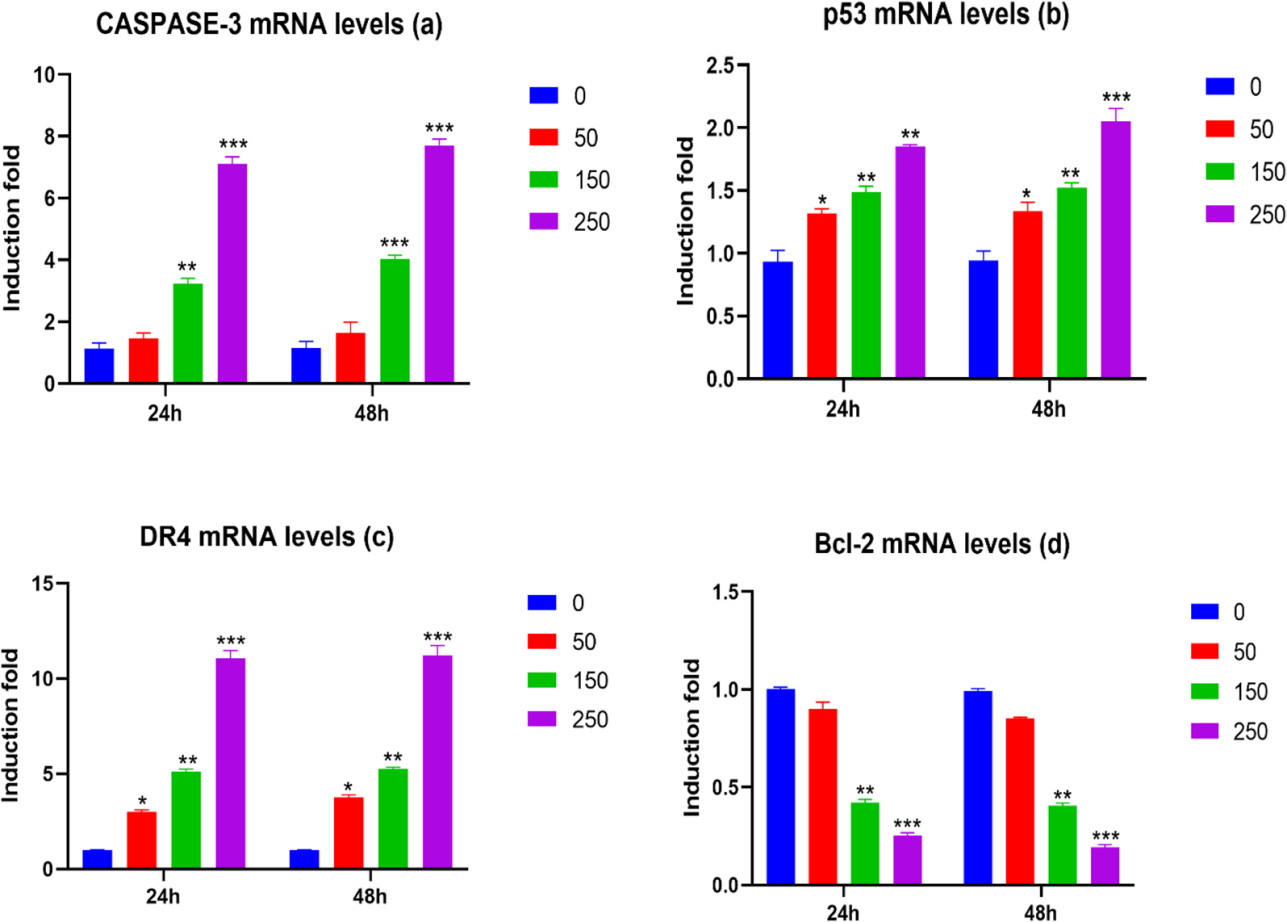
Effect of basil (*Ocimum basilicum L.*) blossoms aqueous extract on apoptotic markers *Caspase-3* (**a**), *p53* (**b**), *DR4* (**c**), and *Bcl-2* (**d**) mRNA levels in MCF7 cells were put under treatment for 24 and 48 h with different groupings of basil (*Ocimum basilicum L.*) blossoms watery extract (0, 50, 150, and 250 µg/mL). From that point, the mRNA levels of *Caspase-3, p53, DR4, *and* BcL2* were evaluated utilizing RT-PCR and standardized to *β-actin* housekeeping gene. Values were recorded as means ± SEM ± SEM (n = 5) of three tests. ***P < 0.001, **P < 0.01, *P < 0.05 contrasted with control (0 µg/mL)

To look at whether the inhibitory impact of (*Ocimum basilicum L.*) blossoms aqueous extract on MCF7 cells expansion and development is an apoptotic-interceded treatment, we decided the limit of (*Ocimum basilicum L.*) blossoms aqueous extract to regulate the outflow of anti-apoptotic and apoptotic genes. For this reason, MCF7 cells were treated for 24 and 48 h with expanding the concentrations of (*Ocimum basilicum L*.) blossoms aqueous extract (0, 50, 150, and 250 µg/mL), as dictated by the outcomes in cell suitability (Fig. 7), from there on *Bcl2*, *Caspase-3*, *p53* and *DR4* mRNA expression levels were controlled by *β-actin*. Figure 7 shows that (*Ocimum basilicum L.*) blossoms aqueous extract essentially actuated *p53*, *caspase-3* and *DR4* mRNA expression levels in a focus subordinate way (Fig. 7a–c). The greatest enlistment was seen at the most elevated fixations tried (50, 150 and 250 µg/mL) in *p53* and *DR4* mRNA expression levels and at (150 and 250 µg/mL) in *caspase-3* mRNA expression levels, respectively. Interestingly, a huge decrease was seen in *Bcl2* mRNA levels because of basil blossoms aqueous extract treatment (Fig. 7d) at the most noteworthy fixation tried (150 and 250 µg/mL).

### Effect of basil (*Ocimum basilicum L.*) blossoms aqueous extract on *Caspase-3* Activity in MCF7 Cells

**Fig. 8:**
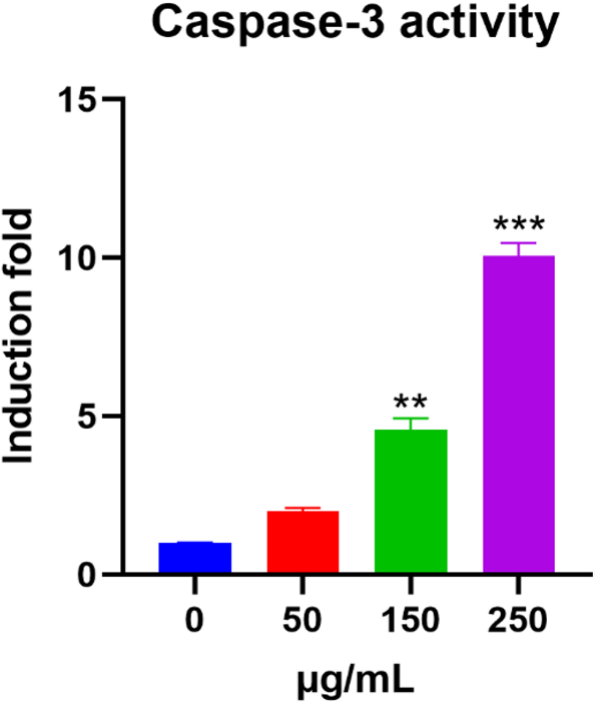
Effect of basil (*Ocimum basilicum L.*) blossoms watery extract on apoptotic *Caspase-3* synergist action in MCF7 cells were treated for 24 h with different convergences of basil (*Ocimum basilicum L.*) blossoms aqueous extract (0, 50, 150, and 250 µg/mL). From there on, *caspase-3* action was resolved calorimetrically utilizing the CaspACE assay. *Caspase3* movement was evaluated by estimating absorbance at a frequency of 405 nm with a plate reader. Data were recorded as means ± SEM (n = 5). ***P < 0.001, **P < 0.01, *P < 0.05 contrasted with control (0 µg/mL)

To inspect whether the *caspase-3* mRNA induction by basil blossoms aqueous extract in MCF7 cells is converted into functional catalytic activities, we analyzed the impact of basil blossoms aqueous extract on *caspase-3* action. For this reason, MCF7 cells were treated for 24 h with expanding convergences of basil blossoms aqueous extract; after which, *caspase-3* activities were resolved calorimetrically utilizing biovision kit in respect to the methods. Figure 8 shows that basil blossoms aqueous extract expanded the activity of *caspase-3* action specifically at higher concentrations, 250 and 150 µg/mL by roughly 10- and 5-folds, individually.

### Assessment the Transcription Inhibitor (Act-D) on the Induction of *Caspase-3* mRNA by basil (*Ocimum basilicum L.*) blossoms aqueous extract in MCF7 Cells

To additionally explore whether the expansion in *caspase-3* mRNA by basil blossoms aqueous extract in MCF7 cells is ascribed to an expansion in the de novo RNA synthesis, MCF7 cells were treated for 24 h with basil blossoms watery extract with the most elevated portion because of its critical impact on other different parameters (250 µg/mL) in the nearness or nonappearance of 10 μg/mL actinomycin D (Act-D), an RNA synthesis inhibitor. *Caspase-3* mRNA was then evaluated by RT-PCR. If basil blossoms watery extract expanded the measure of *caspase-3* mRNA through expanding its de novo RNA synthesis under these conditions, we were relied upon to watch a decline in the substance of *Caspase-3* mRNA after the restraint of its RNA synthesis.

**Fig. 9:**
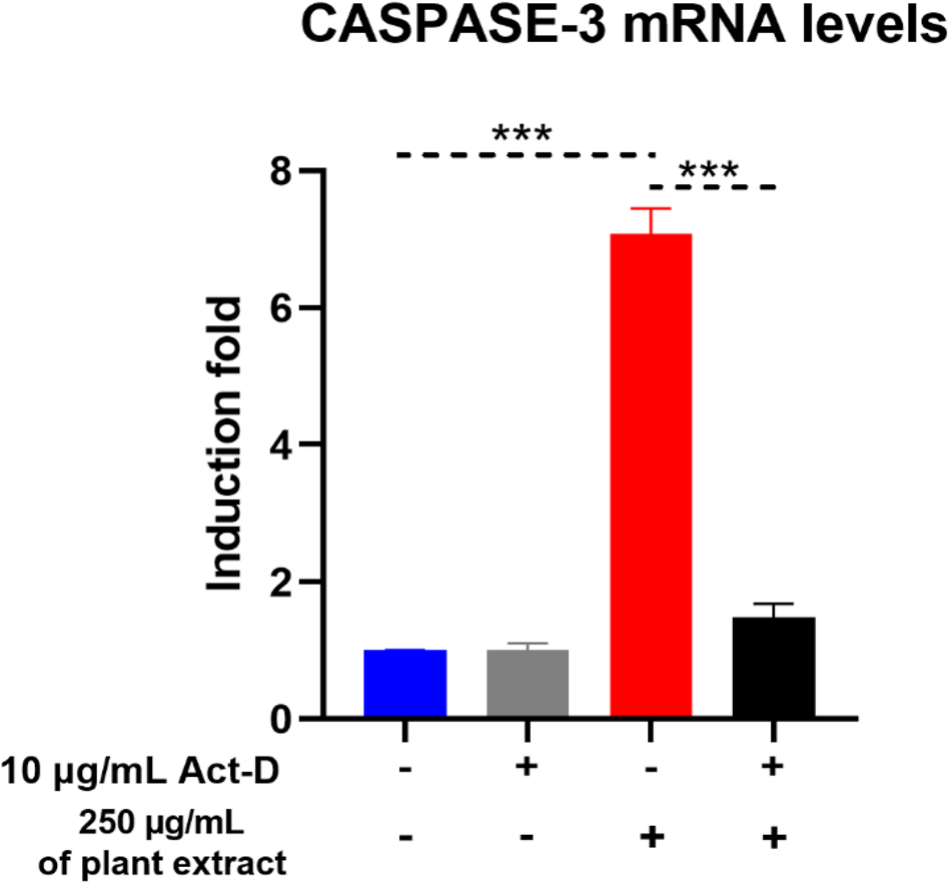
Effect of RNA synthesis inhibitor Act-D on the acceptance *Caspase-3* movement by basil (*Ocimum basilicum L*.) blossoms watery extract in MCF7 cells were treated with 10 μg/mL Act-D, a RNA combination inhibitor, 30 min before exposure to basil roses aqueous extract (250 µg/mL) for extra 24 h. The measure of *Caspase-3* mRNA was evaluated utilizing RT-PCR and standardized to *β-actin* housekeeping quality. Data were recorded as means ± SEM (n = 10) of three tests. ***P < 0.001, **P < 0.01, *P < 0.05 contrasted with same treatment without Act-D

As it appears in Fig. 9, the outcomes demonstrating that Act-D cell pretreatment did not essentially modify the constitutive expression of *Caspase-3* mRNA when contrasted with untreated cells. Nonetheless, the of *Caspase-3* mRNA induction by basil blossoms aqueous extract for 24 utilizing the most noteworthy effective doses (250 µg/mL) was abrogated by Act-D, proposing that basil blossoms aqueous extract expands the *Caspase-3* mRNA level by expanding its de Novo RNA synthesis.

## Discussion

The outcomes from the phytochemical subjective examination of basil (*Ocimum basilicum L.*) blossoms aqueous extract demonstrated that most of the credited bioactivty, for example, cancer prevention agents and anticancer activities of basil blossoms aqueous extract has been ascribed to its secondary and primary metabolite composition [18–20]. Presumably, basil (*Ocimum basilicum L.*) blossoms aqueous extract content recorded higher TFC and TPC (34.25 and 186.31 mg/g) separately, which surpassed what recently detailed by [7, 10, 30]. The aqueous extract was tested particularly for its phenolic composition via elite aqueous chromatography before continuing to cancer prevention agent and anticancer effect assessment. Also, the presence of gallic, quercetin and caffeic compounds in basil (*Ocimum basilicum L.*) blossoms extracts were accounted for in [29], caffeic corrosive and its subsidiaries, for example, rosmarinic corrosive was accounted for as a solid cell reinforcement constituents of basil in [18, 19]. The present investigation gives, as far as anyone is concerned, the principal robotic proof for the capacity of basil (*Ocimum basilicum L.*) blossoms aqueous extract to fundamentally restrain the development and multiplication of breast MCF7 malignant growth cells through apoptotic and oxidative stress intervened systems. Right now, systems basic basil (*Ocimum basilicum L*.) blossoms extricate prompted cell development hindrance and apoptosis were estimated on the accompanying points of view: (a) the development and expansion, (b) mitochondrial movement and glucose take-up inhibition, (c) caspase-3 activity, apoptotic genes and oxidative stress activation, (d) intracellular ROS accumulation, and (e) the job of the Transcription Inhibitor, Act-D, on the Induction of Caspase-3 mRNA by basil (*Ocimum basilicum L.*) blossoms aqueous extract in MCF7 Cells. Significantly high ROS levels in mitochondria can result in free radicals’ attacks on membrane phospholipids that go before mitochondrial film depolarization. Mitochondrial depolarization, viewed as an irreversible advance in apoptosis, triggers a course of caspases [7]. In the current investigation, the µg/mL of basil (*Ocimum basilicum L.*) blossoms aqueous extract treatment effectively brought about the synthesis of ROS [30]. The improvement of ROS creation prompted expanded apoptosis occasions (Fig. 2). Provided that mitochondrial morphology influences imbalances in energy and is ceaselessly changed via fission and fusion events, tight coordination betwixt inter-organelle interactions and mitochondrial dynamics is vital. Mitochondrial splitting outcomes in a disabled insulin-subordinate glucose take-up [22, 28–30]. Apoptosis is a firmly controlled procedure heavily influenced by a few flagging pathways, for example, mitochondrial pathways and caspases [22, 27–29]. Especially, caspase-3 is a significant pro-apoptotic protein inside the intrinsic and extrinsic apoptotic pathways [25, 27]. Caspase-3 activation assumes a focal role in the inception of apoptosis, which requires the actuation of initiator caspases, for example, caspase—9 or 8 or, because of proapoptotic signals [30]. Apoptosis induction with ROS generation by malignant growth chemoprotective agents, for example, doxorubicin [24–26, 30], incites disease cell passing as well as purposes DNA harm and genomic insecurity [16, 30]. Nevertheless, a large portion of these malignant growth chemoprotective treatments are cytotoxic and their utilization is related to toxicities. Thus, the generation of new chemopreventive specialists ready to repress cell expansion and actuate apoptosis in malignant growth cells however with less or no reactions is significant and foreseen. Along these lines, to display the in vivo circumstance, human breast malignancy MCF7 cell lines were utilized in the present examination to anticipate human reactions to basil blossoms aqueous extract by researching the limit of this extract to hinder MCF7 cells development and expansion and investigate the job of apoptosis in basil (*Ocimum basilicum L.*) blossoms extricate—interceded impact.

Also, we have surveyed the potential anticancer impacts of basil blossoms extract with high flavonoids and phenolic substances contrasted with other published investigations that hot water solvent at high temperature or alcoholic solvents [12, 13], subsequently basil blossoms extract stifle the development and multiplication and initiate apoptotic and oxidative impacts in MCF7 cells utilizing basil blossoms aqueous extract was separated at low temperature (0 °C/6 h). In any case, there is past examinations announced that the dull purple basil extract bear more remarkable anticancer and antioxidant activities compared to leave extract [28, 31].

## Conclusion

In conclusion, our findings showed that the aqueous extract of basil (*Ocimum basilicum L*.) blossoms were gathered from Abha, Saudi Arabia and extracted at low temperature (0 °C) resulted in noticeable effects of basil blossoms aqueous extract against cancer growth and oxidant activities. Thus, these anti effects have been ascribed to the presence of high concentrations of flavonoids and phenolic compounds bearing more effective anticancer and antioxidant agents compared to another aqueous extract (using boiled water solvent) and alcoholic extracts.

## Data Availability

All relevant data are within the paper.

## Abbreviations

Act-D: Actinomycin D
ATP: Adenosine triphosphate
Bcl2: B-cell lymphoma 2
cDNA: Complementary DNA
Ct: Cycle threshold
DCF: Dichlorofluorescein
DCF-DA: Dichlorofluorescein diacetate
DNA: Deoxyribonucleic acid OR a double-stranded RNA molecule
DR4: Death receptor 4
g/L: Gram/litter
HO-1: Heme oxygenase-1
HPLC: High-performance liquid chromatography
IC50: The concentration of treatment which inhibited 50% cell viability for MCF7 cells
MCF7: Human breast cancer cell lines
mL/L: Millileter/litter
mRNA: Messenger RNA
NQO1: NAD(P)H Quinone Dehydrogenase 1
PBS: Phosphate-buffered Saline
p53: Phosphoprotein p53 (tumor suppressor)
RNA: Ribonucleic acid OR a single-stranded RNA molecule
ROS: Reactive Oxygen Species
RT-PCR: Real-Time Polymerase chain reaction
TFC: Total flavonoids content
TPC: Total phenolic content
μg/mL: Microgram/millileter
μL: Microliter
